# Spontaneous Tumor Lysis Syndrome in an Adenocarcinoma of Unknown Origin

**DOI:** 10.7759/cureus.12169

**Published:** 2020-12-19

**Authors:** Joshua A Kalter, Jamie Allen, Yuchen Yang, Tyler Willing, Elizabeth Evans

**Affiliations:** 1 Emergency Medicine, University of South Florida Morsani College of Medicine, Tampa, USA; 2 Emergency Medicine, Lehigh Valley Health Network, Allentown, USA

**Keywords:** tumor lysis, adenocarcinoma

## Abstract

Spontaneous tumor lysis syndrome (STLS) is a rare oncologic emergency caused by massive cancer cell lysis or necrosis without a precipitating factor. Although tumor lysis syndrome (TLS) is most commonly associated with hematologic malignancies, a small number of cases in solid tumor malignancies have been reported. We present a case of spontaneous tumor lysis syndrome in a 77-year-old female with a widely metastatic, poorly differentiated adenocarcinoma of unknown origin. She presented in distributive shock, and laboratory testing at admission revealed acute renal failure, high anion gap metabolic acidosis, hyperuricemia, hyperkalemia, hyperphosphatemia, and hypocalcemia. Rasburicase and continuous renal replacement therapy were initiated, however, her condition deteriorated. Treatment was withdrawn and she died four days after admission.

## Introduction

Tumor lysis syndrome (TLS) is an oncologic emergency commonly caused by the challenge of hematologic malignancies with rapid cell turnover or high cancer load with cytotoxic chemotherapy [1]. Less commonly, this can occur in solid malignancies as well as from radiation, corticosteroids, interferon, and tamoxifen treatment [2]. This severe complication results from the release of cellular nucleic acids and electrolytes resulting in elevated serum uric acid, phosphate, and potassium, as well as decreased calcium [1,3]. These metabolic abnormalities can lead to cardiac arrhythmia, seizures, renal injury, multi-organ failure, and death [1]. Diagnosis is made with the Cairo-Bishop classification system either clinically or through laboratory studies [3].

Rarely, TLS can occur spontaneously without preceding intervention. Occurrences are rare with one retrospective study of patients with acute renal failure found that spontaneous TLS was the culprit of 1.1% of cases [4]. Spontaneous TLS (STLS) is most commonly associated with hematologic malignancies [4-6], but a small number of cases have resulted from metastatic solid malignancies such as hepatocellular carcinoma [7], poorly differentiated adenocarcinoma of unknown origin [8-17], cholangiocarcinoma [9], melanoma [10], small cell neuroendocrine carcinoma [11], colon adenocarcinoma [12], gastric cancer [13], prostate adenocarcinoma [14], lung adenocarcinoma [15], and pheochromocytoma [16]. We present a case of an STLS from a patient with an occult undifferentiated adenocarcinoma of unknown origin. 

## Case presentation

**Figure 1:**
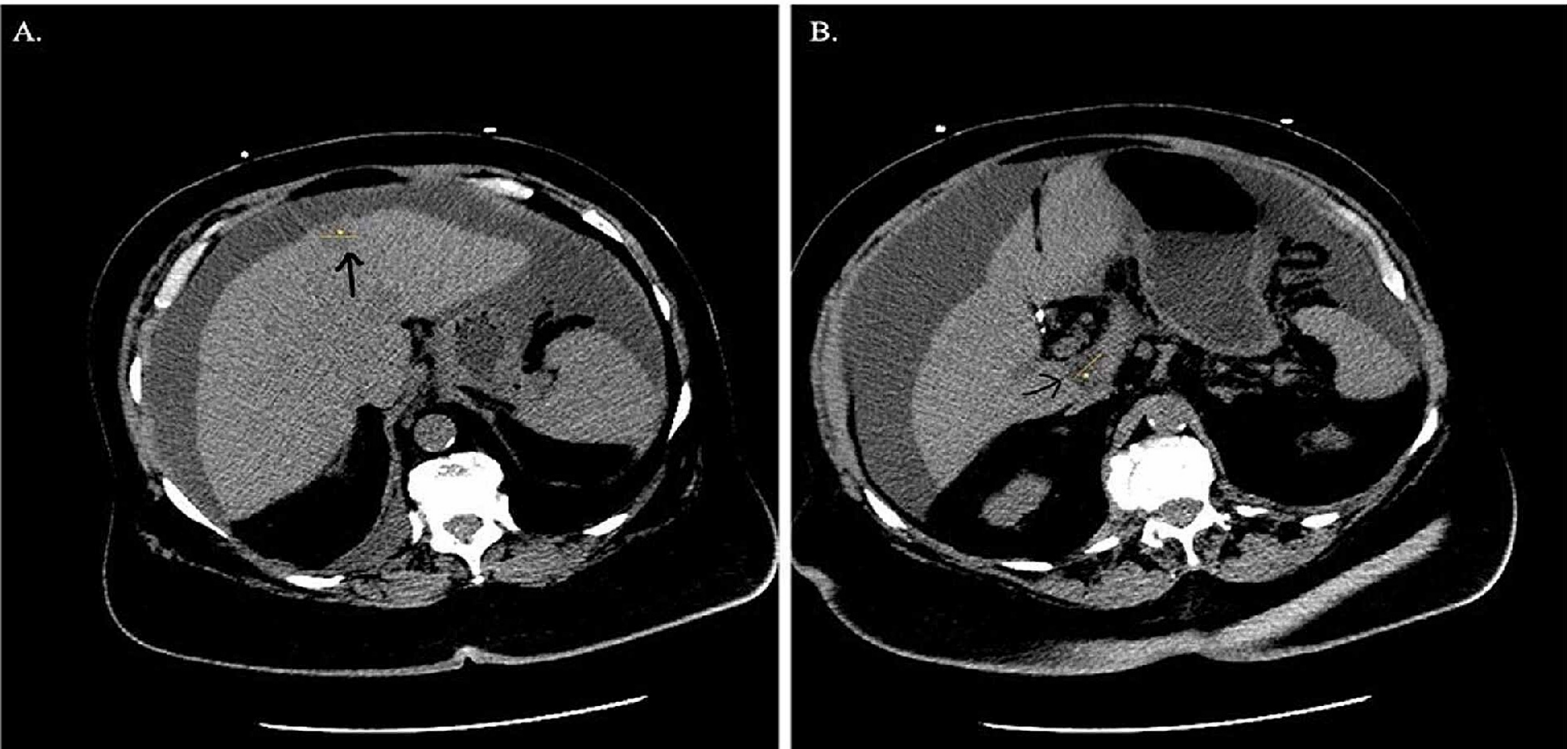
Abdominal CT scan showing hepatic (A) 17.5 mm hypodense segment 4 lesion and (B) 21 mm hypodense segment 1 lesion

A 77-year-old Caucasian female with a past medical history of recurrent breast cancer, hypertension, hyperlipidemia, asthma, diabetic foot ulcer, and osteoporosis presented to the emergency department with dyspnea on exertion, fatigue, difficulty swallowing, and a 10-pound weight loss over several weeks. Her medication list included: aspirin (81 mg daily), vitamin D3 (5000U daily), glucosamine/chondroitin (2 tablets/day), insulin detemir 70u subq, lisinopril (20 mg daily), multivitamin capsule daily, omega-3 fatty acids-vitamin E (1000 mg daily), and silver sulfadiazine 1% cream. Her vital signs were: blood pressure 93/48, pulse 101, temperature 97.9°F (36.6°C) (temporal), respiratory rate 20 and her SpO2 was 97% on room air. Her physical examination was significant for tachycardia; abdominal distension (ascites) without tenderness, rebound, guarding, or fluid wave; and she had dry skin. The rest of the examination was normal. Her laboratory examination revealed a white blood cell count of 16.9 K/cmm (4.0-10.0 K/cmm), BUN of 71 mg/dL (7-18 mg/dL), creatinine of 1.93 mg/dL (0.40-1.00 mg/dL), albumin of 2.5 g/dL (3.5-4.8 g/dL), and a calculated GFR of 25 mL/min/1.73 m^2^ (>60 mL/min/1.73 m^2^). There was a concern for COVID-19 due to her respiratory symptoms and her presentation during the global COVID-19 pandemic. Abdominal and chest CT scan confirmed ascites in addition to right axillary lymphadenopathy, scattered bilateral pulmonary nodular densities, a subcapsular 17.5 mm hypodense liver segment 4 lesion, and a 21 mm hypodense segment 1 lesion that was suspicious for neoplasm (Figure 1), as well as bilateral cystic adnexal masses. The patient was admitted for acute kidney injury, as well as for the investigation of her abdominal mass.

**Figure 2:**
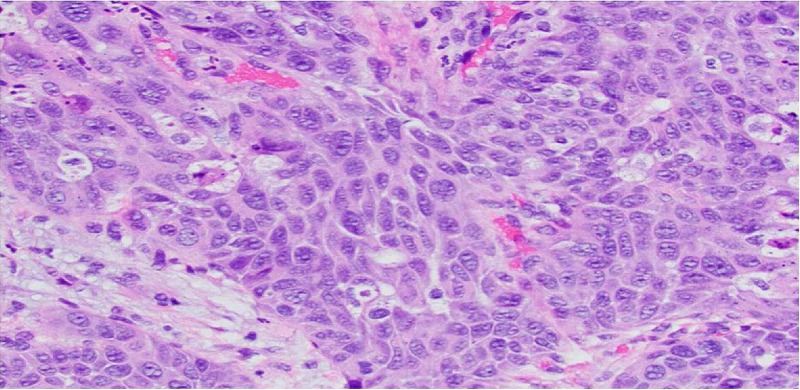
High grade poorly differentiated carcinoma with squamous and glandular features, liver core biopsy x40

While inpatient, she underwent an abdominal paracentesis and the cytology showed atypical glandular cells consistent with metastatic adenocarcinoma. Immunostaining was positive for MOC31, BerEp4, GATA3, and was negative for CK5/6, BRST2, and calretinin. Her cytology was initially believed to be metastatic breast cancer, however, immunostaining was negative for ER, PR, and HER-2/neu. Tumor marker labs and liver biopsy suggested a non-breast malignancy (Figure 2). Tumor markers showed CA 19-9 of 17649, Ca-125 of 482, CEA of 10.2, AFP of 1.2, and Ca 15-3 of 33. An ultrasound-guided liver biopsy was obtained, and the biopsy was positive for metastatic adenocarcinoma. Core biopsy of the liver revealed high grade poorly differentiated carcinoma with both squamous and glandular features (Figure 2). Immunoperoxidase evaluation was consistent with this diagnosis but, the primary malignancy site was indeterminate. Staining was positive for CK7, p40, GATA3, CA19.9, CA125, negative for CK20, CDX2, TTF1, WT1, and PAX8, and inconclusive for BRST2. The tumor was GATA3 positive which was likely due to p40 positive squamous differentiation. Subsequently, CancerTYPE ID, molecular cancer classification test, Biotheranostics Inc. was performed demonstrating a 90% probability of pancreatobiliary origin.

Twenty-two days after initial presentation to the ED, the patient was noted to be hypotensive when she arrived for scheduled port placement. She was sent to the emergency department where she complained of dyspnea and swelling of her left lower extremity. Laboratory work revealed a hemoglobin of 9.4 (11.5-14.5 g/dL), PT of 15.8 (12.0-14.6 sec), and pertinent findings in Table 1. Due to her elevated troponin and swollen leg, a Doppler ultrasound was obtained and revealed a deep venous thrombosis (DVT). She was admitted to the intensive care unit on multiple vasopressors. Hematology and Oncology were consulted who recommended rasburicase for hyperuricemia. Continuous renal replacement therapy (CRRT) was initiated.

**Table 1:** Laboratory Work Results BUN: blood urea nitrogen

Laboratory Work Results								
Day in Hospital	BUN (7-25 mg/dL)	Cr (0.4-1.1 mg/dL)	K+ (3.5-5.2 mmol/L)	Ca2+ (8.5-10.1 mg/dL)	Phosphorous (2.3-4.6 mg/dL)	Uric Acid (2.4-6.0 mg/dL)	Anion Gap (8-12)	Troponin
1	154	6.99	5.3	6.6	9.1	14.6	25	0.09

The patient’s renal function normalized and her hyperuricemia corrected with rasburicase. CT angiogram of her chest was negative for pulmonary embolism. Her echocardiogram was unremarkable. Septic workup including blood cultures, respiratory viral panel, and urine culture was negative. Unfortunately, her overall condition deteriorated. After a family discussion, comfort care was initiated and the patient expired on hospital day four.

## Discussion

Tumor lysis syndrome is a serious complication for hematology/oncology patients, which is characterized by hyperkalemia, hyperuricemia, hyperphosphatemia, and hypocalcemia [1,3]. These metabolic derangements have serious complications that precipitate renal failure, seizures, cardiac arrhythmia, and death [1]. Spontaneous tumor lysis syndrome is a rare manifestation of this complication without any preceding chemotherapeutic intervention and is most commonly associated with hematologic malignancies [4-16]. Prompt management of TLS with aggressive hydration, allopurinol or rasburicase to decrease uric acid, dialysis or glucose with insulin or beta-agonists to decrease potassium, and calcium replacement are important interventions to correct metabolic abnormalities [1].

Several factors increase the risk of TLS development, such as high tumor burden, preceding renal injury, malignant liver and spleen infiltration, high proliferative rate, and sensitivity to chemotherapy [1]. Though spontaneous tumor lysis syndrome is associated with hematologic malignancies, case reports in solid malignancies appear to show a common feature, namely a high tumor burden in the liver. Our patient shared this feature [7-16]. Additionally, the preceding acute kidney injury in our patient may have contributed to the precipitation of STLS. While our case is not the first case of STLS by an undifferentiated adenocarcinoma of unknown origin, Saini et al [8] reported a mass that stained positive for CK7, CK20, mucicarmine, and villin, but negative for ER, mammaglobin, TTF-1, napsinA, CDX2, p63, calretinin and hepatocyte antigen that was suggestive of a non-colorectal gastrointestinal origin. This staining differs from our patient’s adenocarcinoma, and the group did not report serum tumor markers [8]. 

Upon retrospective chart review, it was noted that our patient was given a four-day burst of oral dexamethasone starting seven days prior to her presentation to our ED with hypotensive shock. While both high and low-dose dexamethasone and prednisone have been noted to cause TLS in patients with hematologic malignancies, an extensive literature search found one case of steroid-induced TLS in a patient with a solid tumor (hepatocellular carcinoma). Though the seven-day period between initiation of steroids and presentation with TLS makes this a less likely etiology, possible TLS should be considered with steroid initiation in a patient with metastatic disease.

## Conclusions

Spontaneous tumor lysis syndrome (STLS) is a rare oncologic emergency caused by massive cancer cell lysis or necrosis without a precipitating factor. Even more rare is for cases to originate in patients with solid tumors. This case emphasizes that it is an important diagnosis to consider in patients with known cancer or those at risk for having undiagnosed cancer.
